# ToxDBScan: Large-Scale Similarity Screening of Toxicological Databases for Drug Candidates

**DOI:** 10.3390/ijms151019037

**Published:** 2014-10-21

**Authors:** Michael Römer, Linus Backert, Johannes Eichner, Andreas Zell

**Affiliations:** Center of Bioinformatics Tuebingen (ZBIT), University of Tuebingen, Tübingen 72074, Germany; E-Mails: backert@informatik.uni-tuebingen.de (L.B.); johannes.eichner@uni-tuebingen.de (J.E.); andreas.zell@uni-tuebingen.de (A.Z.)

**Keywords:** TG-GATEs, DrugMatrix, carcinogenic, toxicogenomics, mRNA, microarrays, drug discovery, visualization, similarity, web application

## Abstract

We present a new tool for hepatocarcinogenicity evaluation of drug candidates in rodents. ToxDBScan is a web tool offering quick and easy similarity screening of new drug candidates against two large-scale public databases, which contain expression profiles for substances with known carcinogenic profiles: TG-GATEs and DrugMatrix. ToxDBScan uses a set similarity score that computes the putative similarity based on similar expression of genes to identify chemicals with similar genotoxic and hepatocarcinogenic potential. We propose using a discretized representation of expression profiles, which use only information on up- or down-regulation of genes as relevant features. Therefore, only the deregulated genes are required as input. ToxDBScan provides an extensive report on similar compounds, which includes additional information on compounds, differential genes and pathway enrichments. We evaluated ToxDBScan with expression data from 15 chemicals with known hepatocarcinogenic potential and observed a sensitivity of 88%. Based on the identified chemicals, we achieved perfect classification of the independent test set. ToxDBScan is publicly available from the ZBIT Bioinformatics Toolbox.

## 1. Introduction

Developing new drugs is a very cost-intensive process. Estimates of the overall cost for developing Food and Drug Administration (FDA) approved drugs range from $160 million to $1.8 billion for one drug, based on success rates of only 12% to 23% for drugs entering the clinical phase [1]. Low success rates in combination with high requirements for approval by the FDA or similar agencies lead to the immense costs per approved drug. Depending on the estimate, between 40 and 65 percent of the total cost is spent during the preclinical phase [1]. Animal studies are required prior to approval for clinical studies. These animal studies are expensive, both in terms of required resources (*i.e.*, animals, researchers, chemicals), as well as time. While clinical trials are generally more expensive than preclinical trials, the success rate is much lower for preclinical trials. Therefore, a larger number of preclinical trials is required per approved drug, which leads to high costs accumulating in the preclinical phase.

Drug candidates with genotoxic effects are identified early in the preclinical phase with genotoxicity assays, e.g., the Ames test [2]. However, carcinogenic effects can also arise irrespective of genotoxic events, e.g., by inhibition of apoptosis or initiation of proliferation [3]. Currently, no approved short-term assays are available for non-genotoxic carcinogenicity. The current gold standard in the preclinical assessment of non-genotoxic carcinogenicity is the two-year rodent assay [4]. During this assay, a group of rodents, typically rat or mice, is treated with the drug candidate in a multitude of the estimated human dosagaes (see ICH Safety Guidelines S1A-S1C and OECD Test Guideline 451). The treated group is compared with a non-treated control group to identify a potential increase in cancer incidents, e.g., by histopathology. This process is not only cost-intensive, but can lead to the late discovery of the carcinogenic effects of the drug candidate. These failures late in the preclinical phase contribute to the low success rate and are particularly expensive [5].

The field of toxicogenomics uses computational biology approaches to investigate toxicological questions, such as carcinogenicity prediction for drug candidates. This includes *in silico* approaches, e.g., quantitative structure-activity relationship (QSAR) models [6], as well as approaches that combine high-throughput methods, such as microarrays or next-generation sequencing with computational analysis [7]. The combination of short-term rodent assays with machine learning has been shown to be able to predict the outcome of the two-year rodent assay [7]. This toxicogenomics approach uses microarray data obtained from the treated and control animals after one, two or four weeks, or even a longer duration of treatment with the drug candidate.

The problems of these studies are the small sample size, due to budget or time restrictions, and the large diversity of potential modes of action (MOAs) observed for non-genotoxic carcinogens. Whereas DNA damage response and p53 signaling can be observed for most genotoxic carcinogens, non-genotoxic carcinogens act through several distinct mechanisms, e.g., chronic cell injury, immunosuppression, increased secretion of trophic hormones or altering receptor activity [3]. During the last decade, the problem posed by non-genotoxic carcinogens led to the development of two large databases that are publicly available: Open TG-GATEs [8] and DrugMatrix [9]. These databases allow a more comprehensive analysis of MOAs of non-genotoxic carcinogens. To our knowledge, no tool exists that allows the analysis of both databases. Most studies that investigated toxicogenomics approaches focused on established machine learning methods that are applied to expression profiles obtained from specific microarrays [7]. Therefore, it is difficult to construct prediction systems for new expression profiles that were obtained by different researchers under different conditions.

In this paper, we present ToxDBScan, which is an easy interface for the information included in these databases. ToxDBScan enables researchers to quickly identify compounds that show similar perturbations on the gene expression level in the liver of male rats. Compatibility across available microarray platforms is provided through the abstraction of array-specific probe set identifiers to gene symbols. In addition, ToxDBScan performs pathway enrichment analyses against the KEGG database [10]. The ToxDBScan web application is freely available from the ZBIT Bioinformatics Toolbox [11]. Because only the up- and down-regulated genes are required as input for the web application, no confidential data needs to be uploaded in order to perform analyses. This allows a quick and easy identification of potentially similar compounds for further mechanistic analysis, assessment of their hepatocarcinogenic potential or mode of action discovery.

## 2. Results and Discussion

### 2.1. Gene Fingerprint Extraction

**Figure 1:**
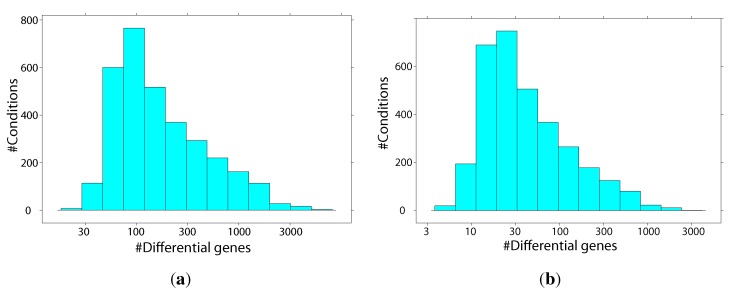
Gene fingerprint sizes for different intensity ratio thresholds. (**a**) Low threshold, 1.5-fold deregulation; (**b**) high threshold, two-fold deregulation.

Gene fingerprints were extracted for each condition based on the intensity ratio (treated to control animals) with two thresholds: 1.5-fold and two-fold deregulation. Figure 1 shows the distribution of gene fingerprint sizes. At least one deregulated gene was identified for each threshold in all conditions.

For the less conservative 1.5-fold deregulation threshold, the gene fingerprint size ranges from 23 to 6525 genes, with a median size of 131 genes. Gene fingerprint sizes were smaller for conditions from TG-GATEs, with a median size of 111 genes, compared to a median size of 603 genes for DrugMatrix conditions. For the stricter two-fold deregulation threshold, gene fingerprint sizes range from 5 to 3224 genes, with a median size of 32 genes. Again, gene fingerprint sizes were smaller for TG-GATEs conditions, with a median size of 27 genes, compared to a median size of 152 genes for conditions from DrugMatrix. This difference may be a result of the higher dose levels administered in DrugMatrix experiments.

### 2.2. Identification of Similar Conditions

For each chemical in the evaluation dataset (see Table 1), we used our similarity score to extract the most similar conditions from the combined TG-GATEs and DrugMatrix databases. The extracted conditions were compared to the evaluation chemicals based on genotoxicity and carcinogenicity information.

**Table 1:** Chemicals used for evaluation. Male Wistar rats were treated with the chemicals each day for up to 14 days. For each chemical, the Chemical Abstracts Service (CAS) registry number, dosing time and dose is listed, as well as the short name that is used in the tables and figures. The last column lists the databases that contain the test compound (DM = DrugMatrix, TGG = TG-GATEs). Adapted from Römer *et al.* [12].

Compound	Short Name	CAS Number	Dosing Time (day)	Dose (mg/kg/day)	Contained in
Genotoxic carcinogens (GCs)					
Direct Black 38	CIDB	1937-37-7	7	146	-
Nitrosodimethylamine	DMN	62-75-9	7	4	DM
Non-genotoxic carcinogens (NGCs)					
Piperonyl butoxide	PBO	51-03-6	3	1200	-
Methyl carbamate	MCA	598-55-0	14	400	-
Dehydroepiandrosterone	DHEA	53-43-0	14	600	-
Methapyrilene	MP	135-23-9	14	60	TGG, DM
Thioacetamide	TAA	62-55-5	7	19.2	TGG, DM
Diethylstilbestrol	DES	56-53-1	3	10	DM
Wy-14643	WY	50892-23-4	3	60	TGG, DM
Acetamide	AAA	60-35-5	14	3000	TGG
Ethionine	ET	67-21-0	14	200	TGG
Cyproterone acetate	CPR	427-51-0	14	100	DM
Phenobarbital	PB	50-06-6	14	80	TGG, DM
Non-hepatocarcinogens (NCs)					
Cefuroxime	CFX	55268-75-2	14	250	-
Nifedipine	NIF	21829-25-4	14	3	TGG

Ten of the 15 chemicals in the evaluation set are contained in either TG-GATEs, DrugMatrix or both. These 10 substances included eight non-genotoxic carcinogens (NGCs), one non-hepatocarcinogen (NC) (nifedipine, NIF) and one GC (nitrosodimethylamine, DMN). For five of these eight NGCs (acetamide (AAA), ethionine (ET), methapyrilene (MP), phenobarbital (PB) and thioacetamide (TAA)) an experiment with the same substance was returned as the best hit (see Figure S1). The remaining three NGCs (cyproterone acetate (CPA), diethylstilbestrol (DES) and Wy-14643 (WY)) were placed second in the returned list of similar experiments, due to the existence of related NGCs for which higher similarity scores were observed (see Figure S1).

**Figure 2:**
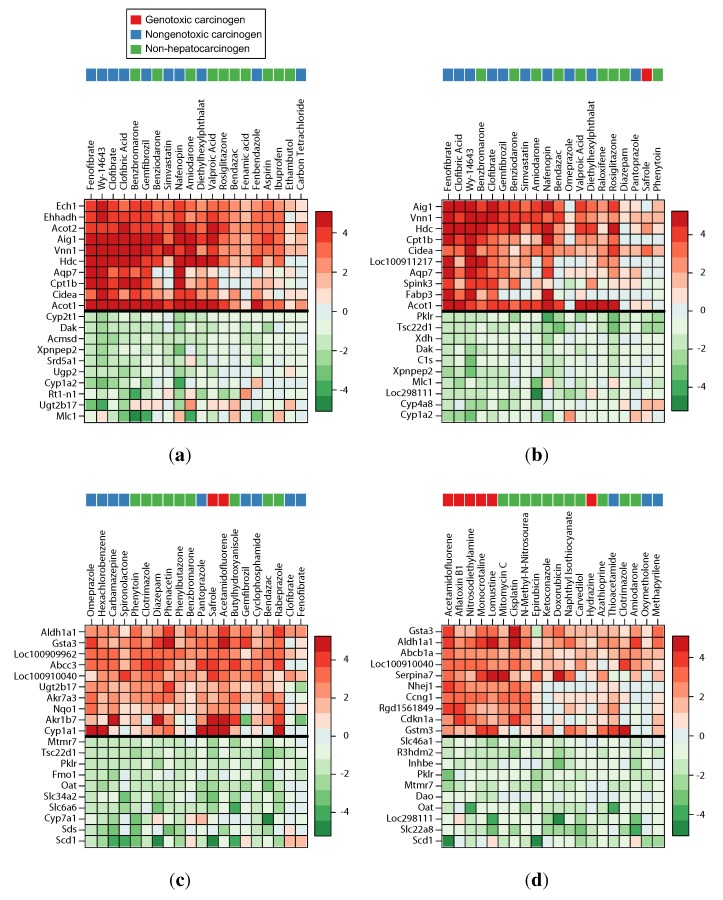
Gene expression heat maps of similar compounds. For selected test chemicals, we extracted the most similar chemicals included in either TG-GATEs or DrugMatrix. Each column corresponds to a chemical that was identified as similar. The chemicals are sorted from left to right by descending similarity score. The heat maps show the log2 fold change of 20 selected genes from the gene fingerprints of the test chemical. Genes above the black line are upregulated at least 1.5-fold in the test chemical, and genes below are downregulated, respectively. Genes were selected based on average expression in the identified chemicals. The color bar above the chemical name indicates the hepatocarcinogenicity annotation, and the legend is shown in (**a**). (**a**) Wy-14643 (NGC); (**b**) dehydroepiandrosterone (NGC); (**c**) piperonyl butoxide (NGC); (**d**) C.I Direct Black (GC).

The identification of similar NGCs also allows a mode of action analysis. For instance, WY was found to be most similar to the chemicals, fenofibrate, clofibric acid and clofibrate (see Figure 2a). These are known to activate peroxisome proliferator-activated receptor alpha (PPARa) [13], suggesting a PPARa-related mode of action for WY (as shown by Peraza *et al.* [13]). Fenofibrate, clofibric acid, WY and clofibrate are also among the most similar chemicals for DHEA (see Figure 2b). This may indicate a PPARa-related mode of action for DHEA, as has previously been shown by Mastrocola *et al.* [14].

For PBO, several NGCs were identified as the most similar chemicals: omeprazole, hexachlorobenzene, carbamazepine and spironolactone (see Figure 2c). Three of these chemicals, omeprazole, hexachlorobenzene and carbamazepine, are classified as enzyme inducers [15,16], suggesting an enzyme-inducing mode of action for PBO, as demonstrated by Goldstein *et al.* [17]. Similar results are obtained for other enzyme inducers in the test set, e.g., cyproterone acetate (CPR) and PB. Omeprazole, spironolactone and carbamazepine are found among the most similar compounds for CPR, suggesting enzyme induction as the major MOA, as Schulte-Hermann *et al.* demonstrated [18]. Carbamazepine and hexachlorobenzene are among the most similar compounds for PB, which again suggests enzyme induction as an MOA, as has been shown by Waxman *et al.* [19]. Sulfasalazine, which is classified as an enzyme-inducing NGC by Uehara *et al.* [16], is also among the compounds most similar to PB, but has no associated positive test for hepatocarcinogenicity in CPDB and is therefore considered an NC.

The NGCs, TAA, MP and ET, are considered hepatotoxic oxidative stressors by Uehara *et al.* [16]. For TAA, the most similar compound is MP, but with a low similarity compared to the TAA experiments contained in TG-GATEs. Among the compounds most similar to MP are carbon tetrachloride and TAA, which supports the hepatotoxic MOA, but also the PPARa-activator, gemfibrozil, and the genotoxic compound, hydrazine. For ET, the most similar compounds include TAA and MP, as well as carbon tetrachloride, which is also a hepatotoxic oxidative stressor [16].

The genotoxic compound DMN was not recalled, which may also be due to the different dosage and duration of treatment (10 mg/kg/day for five days in DrugMatrix *vs.* 4 mg/kg/day for seven days in the evaluation dataset). However, nitrosodiethylamine, which is very similar to DMN chemically, was identified as the most similar compound for DMN, along with other GCs. For the second genotoxic chemical, C.I Direct Black (CIDB), the five most similar compounds identified in the databases are all GCs; the highest scoring is acetamidofluorene (see Figure 2d).

This evaluation shows that our similarity score allows the identification of similar compounds to provide leads for mechanistic analysis, carcinogenicity evaluation and mode of action detection.

### 2.3. Threshold Selection

In order to select an appropriate similarity threshold for the compound fingerprints, we determined for each chemical how many conditions with an equal toxicological class are among the five, 10 and 20 nearest neighbors, *i.e*., most similar conditions (see Table 2). The less conservative threshold of 1.5-fold deregulation performs slightly better than the stricter threshold. On average, 4.3 out of the five, 8.0 out of the 10 and 14.4 out of the 20 most similar were treated with a chemical of the same carcinogenicity class. For each chemical in the test set, relative similarity scores *S̃* were computed by dividing the observed similarity score for a certain condition by the maximum similarity score. The percentage of conditions annotated with the same carcinogenicity class in the subset of conditions with a relative similarity score higher than 0.8 and 0.7 was computed (see Table 3). Again, a slightly better performance was observed for the less conservative fold change cutoff. On average, 88% of the identified conditions with a *S̃* > 0.8 were of the same class as the evaluation chemical, while only 80% conditions with matching classes were found for *S̃* > 0.7. Our evaluation with expression profiles from an independent dataset show that our similarity score allows robust identification of compounds with similar genotoxic and hepatocarcinogenic potential. The identification is possible for chemicals that are already in one or both databases, as well as for compounds that are not included in any of the two databases.

**Table 2:** Percentage of correctly identified conditions. The most similar conditions were extracted for each chemical in the evaluation set. The percentage of conditions with the same carcinogenicity class in the five, 10 and 20 most similar conditions was calculated.

Chemical	1.5-Fold Deregulation				2-Fold Deregulation		
	Best 5	Best 10	Best 20		Best 5	Best 10	Best 20
Genotoxic carcinogens							
CIDB	100	100	95		100	90	90
DMN	80	70	50		100	80	75
Non-genotoxic carcinogens							
PBO	100	80	65		80	70	75
MCA	60	60	45		80	60	50
DHEA	100	90	85		100	90	80
MP	80	70	70		80	50	40
TAA	100	80	65		100	70	60
DES	100	100	100		100	100	100
WY	100	90	90		100	100	95
AAA	60	50	35		80	60	50
ET	100	100	100		100	100	85
CPR	80	80	65		60	60	60
PB	80	60	45		20	20	25
Non-hepatocarcinogens							
CFX	100	90	85		60	70	85
NIF	60	80	70		60	70	70
Mean	86	80	71		82	73	70

**Table 3:** Percentage of correctly identified conditions. The most similar conditions were extracted for each chemical in the evaluation set. The percentage of conditions with the same carcinogenicity class and a relative similarity above 0.8 and 0.7 was calculated.

Chemical	1.5-Fold Deregulation			2-Fold Deregulation	
	*S̃* ≥ 0.8	*S̃* ≥ 0.7		*S̃* ≥ 0.8	*S̃* ≥ 0.7
Genotoxic carcinogens					
CIDB	100	100		100	100
DMN	70	48		78	71
Non-genotoxic carcinogens					
PBO	77	66		73	76
MCA	100	100		100	100
DHEA	88	89		100	80
MP	100	100		100	100
TAA	100	100		100	67
DES	100	100		100	100
WY	100	91		100	94
AAA	60	42		67	55
ET	100	100		100	100
CPR	71	57		50	60
PB	100	60		0	20
Non-hepatocarcinogens					
CFX	100	88		33	50
NIF	50	67		100	100
Mean	88	80		80	78

Across all evaluations, the 1.5-fold deregulation threshold led to better results for the similarity search. This may be due to the larger number of genes available for the similarity scoring of the evaluation compounds (median fingerprint size: 269 genes). The smaller fingerprint sizes observed for the higher threshold (see Figure 1) may contain too few specific genes, which are only slightly deregulated. Particularly, for NGCs and NCs, the number of deregulated genes is very small when using the two-fold deregulation threshold, with a median fingerprint size of 53 genes. Based on the above evaluations, we propose using the 1.5-fold deregulation threshold and consider conditions with a relative similarity score *S̃* > 0.8 as likely to share the same class.

### 2.4. Hepatocarcinogenicity Prediction

Above, we proposed using an intensity ratio threshold of 1.5-fold deregulation for gene fingerprint extraction and a relative fingerprint similarity of more than 0.8 to identify similar compounds. To assess the viability of these thresholds, we performed a classification of an independent test set. For each chemical, we extracted gene fingerprints using a 1.5-fold deregulation threshold. These were compared to the database using our similarity score. Conditions with a relative similarity score *S̃* ≥ 0.8 were considered as similar, whereas conditions with *S̃* < 0.8 . The results of the over-representation test are shown in Table 4. The correct class was predicted for all 15 chemicals in the evaluation set.

**Table 4:** Classification results. Similar conditions in TG-GATEs and DrugMatrix were identified by computing the similarity score *S* and selecting conditions with a relative similarity *S̃* > 0.8. Ratios of genotoxic carcinogens (*R*_GC_) and non-genotoxic carcinogens (*R*_NGC_) were computed based on the annotation of the similar conditions. A permutation test (*n* = 100*,* 000) was performed to assess the significance of over-representation of GCs (*p*_GC_) and NGCs (*p*_NGC_). If the *p*-values were significant for *α* = 0.05, the corresponding class was predicted. If no significant enrichment was found for either of the two classes, the test chemical was predicted as non-hepatocarcinogen (NC). Significant *p*-values are printed in bold font.

Chemical	*R*_GC_	*p*_GC_	*R*_NGC_	*p*_NGC_	Prediction
Genotoxic carcinogens					
CIDB	1.00	**0.001**	0.00	1.000	GC
DMN	0.70	**0.008**	0.20	0.615	GC
Non-genotoxic carcinogens					
MP	0.00	1.000	1.00	**0.007**	NGC
TAA	0.00	1.000	1.00	**0.012**	NGC
DES	0.00	1.000	1.00	**<0.001**	NGC
WY	0.00	1.000	1.00	**<0.001**	NGC
PBO	0.00	1.000	0.77	**0.002**	NGC
MCA	0.00	1.000	1.00	**0.017**	NGC
AAA	0.00	1.000	0.60	**0.048**	NGC
DHEA	0.00	1.000	0.88	**<0.001**	NGC
ET	0.00	1.000	1.00	**<0.001**	NGC
CPR	0.00	1.000	0.71	**0.018**	NGC
PB	0.00	1.000	1.00	**0.019**	NGC
Non-hepatocarcinogens					
CFX	0.00	1.000	0.00	1.000	NC
NIF	0.25	0.132	0.25	0.399	NC

### 2.5. Web Application

**Figure 3:**
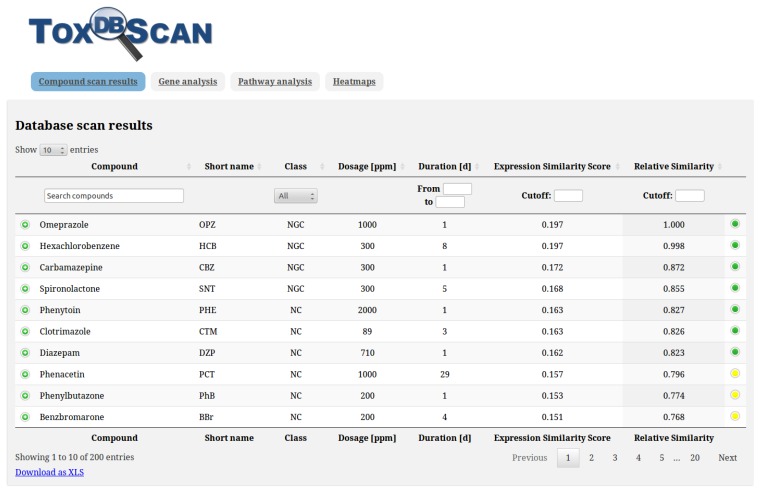
HTML report of the compound similarity scan for PBO. This figure shows the results of the similarity search against TG-GATEs and DrugMatrix. Additional information for each compound can be shown by clicking on the “plus” in the first column of the table. Additional information on the deregulated genes is available from the “Gene analysis” tab at the head of the report. The results of the pathway enrichment analysis against the KEGG database are available from the “Pathway analysis” tab. The “Heat maps” tab shows heat maps of the gene expression in the most similar compounds.

ToxDBScan is available as a web application from the ZBIT Bioinformatics Toolbox [11]. The ZBIT Bioinformatics Toolbox runs on a Galaxy Project web server [20,21,22], which provides a user-friendly and sustainable platform for tools used in scientific research. No local installation is required for running the application. ToxDBScan generates an HTML report, which is shown directly inside the ZBIT Bioinformatics Toolbox. This report includes the results of the database scan for similar compounds, enriched KEGG pathways, as well as information on the NGC-specificity and information content of the deregulated genes (see Figure 3). This information can be used for a mechanistic analysis of the hepatocarcinogenic potential or mode of action detection. The gene fingerprint of the query compound can be compared to the gene expression profiles observed under the most similar conditions by means of a heat map.

Additional information on the database compounds (e.g., CAS number and structure) and KEGG pathways is provided. All reports can be downloaded for further analyses in either tabular format or as a PDF. ToxDBScan requires only the deregulated genes observed in an experiment, which can be provided as either official rat gene symbols (as provided by the Rat Genome Database [23]), Entrez IDs [24], Ensembl IDs [25] or UniProt IDs [26]. Therefore, no confidential data needs to be uploaded, such as the chemical structure, name or experimental details.

### 2.6. Discussion

We have developed a novel approach for similarity scoring of gene expression profiles and applied it to data from TG-GATEs and DrugMatrix, two large-scale toxicogenomics databases. We evaluated our similarity score with an independent evaluation set of gene expression profiles from experiments not included in TG-GATEs and DrugMatrix. The results indicate that our similarity score is able to robustly identify hepatocarcinogenic compounds with similar modes of action. Furthermore, we demonstrated that an accurate prediction of the carcinogenicity class of the evaluation chemicals was possible. All 15 compounds in the evaluation dataset were assigned to the correct class. The similarity score can be used through a web application to identify compounds with a potentially similar mode of action in TG-GATEs and DrugMatrix. The web application, ToxDBScan, is freely available from the ZBIT Bioinformatics Toolbox [11].

The evaluation dataset included 15 chemicals belonging into three carcinogenicity classes: NGCs, GCs and NCs. In our evaluation dataset, three major mechanisms are represented: oxidative stress-mediated hepatotoxicity (TAA, MP, ET), PPARa-induction (WY, DHEA) and enzyme induction (PB, PBO, CPR) [13,14,16,17,18]. Our similarity score robustly identified compounds in TG-GATEs and DrugMatrix that act through the same modes of action as these NGCs. Furthermore, GCs in the databases were identified as most similar to the genotoxic evaluation chemicals (CIDB, DMN). This indicates that our similarity score is a useful tool for the identification of compounds in TG-GATEs and DrugMatrix that act through similar mechanisms, thus providing leads for further analysis of the mode of action.

To assess if our similarity score can be used for the identification of the hepatocarcinogenicity of new drug candidates, we evaluated different intensity-ratio cutoffs and relative similarity thresholds. The best results were obtained with a 1.5-fold deregulation threshold for genes and a 0.8 relative similarity threshold. With these parameters, we observed that 88% of the compounds that were identified as similar have equal hepatocarcinogenic potential. Using these optimal parameters, we performed a classification of the independent evaluation compounds based on the TG-GATEs and DrugMatrix databases. We were able to correctly predict all 15 evaluation chemicals as NGC, GC or NC. This indicates that our similarity score allows the hepatocarcinogenicity evaluation of new compounds based on large databases of compounds with known hepatocarcinogenic potential.

ToxDBScan, a web application that is freely available, was created to allow other researchers to use our similarity score for the identification of similar compounds in TG-GATEs and DrugMatrix. To our knowledge, no other web application is available that offers a similarity search in both TG-GATEs and DrugMatrix. In addition, ToxDBScan is independent of the platform used to identify the deregulated genes, as only the list of up- and down-regulated genes is required to run ToxDBScan. New data can easily be integrated into ToxDBScan to extend the database of expression profiles available for the similarity search. The use of ToxDBScan is not limited to new drug candidates, as demonstrated by the compound, CIDB, in our evaluation set, which is a genotoxic dye. In summary, ToxDBScan offers a unique similarity scoring method for the two largest toxicogenomics databases and may contribute to the implementation of new approaches for the evaluation of the carcinogenic potential of chemicals.

## 3. Experimental Section

### 3.1. Data Resources

The data sources for ToxDBScan are the two largest publicly available toxicogenomics databases: DrugMatrix [9,27] and the Toxicogenomics Project-Genome Assisted Toxicity Evaluation System (TG-GATEs) [8,28]. Hepatocarcinogenicity and genotoxicity annotation was performed using the Carcinogenic Potency Database (CPDB) [29,30].

#### 3.1.1. Carcinogenic Potency Database

The Carcinogenic Potency Database (CPDB) is a publicly available database, which records the outcome of long-term *in vivo* cancer bioassays performed in several organisms. Currently, it contains the outcome of 6540 studies on 1547 chemicals. The carcinogenic potential is listed by the observed cancer site. In addition, the outcome of an auxotroph-based Ames test is contained for many chemicals. For this study, we considered a chemical hepatocarcinogenic if the CPDB contained a positive outcome that was observed in the liver of male rat and genotoxic if a positive outcome of the Ames test was recorded. Compounds that were not tested in the CPDB or that have no distinct associated outcome are annotated as unclassified. Chemicals were classified as genotoxic carcinogens (GC) if they were both hepatocarcinogenic and genotoxic, non-genotoxic carcinogens (NGC) if they were hepatocarcinogenic and not genotoxic or non-hepatocarcinogenic (NC) if no positive carcinogenicity test in male rat liver was recorded in the CPDB (see Table S1).

#### 3.1.2. Toxicogenomics Project-Genome Assisted Toxicity Evaluation System

TG-GATEs is a publicly available toxicogenomics database, which was established by the Japanese government and several Japanese pharmaceutical companies [8,31]. It is available from ArrayExpress through the accession number, E-MTAB-800. TG-GATEs contains gene expression profiles from male Sprague-Dawley rat liver and kidney, as well as cultured human and rat hepatocytes treated with 160 chemicals in either single or repeated dosage settings. For ToxDBScan, all expression profiles from the rat liver were used. Each chemical was administered at three doses and for eight durations, *i.e.*, 3 to 24 h in the single dosage setting and 4, 8, 19 and 29 days in repeated dosage setting. In total, 3528 combinations of chemical, dosage and duration were performed with three replicates each. Three matched controls were profiled for each condition, leading to 14,143 available gene expression profiles. Through CPDB and Uehara *et al.* [16], genotoxicity annotations are available for 123 of the 160 compounds profiled in male rat liver, which translates to 2768 conditions with known hepatocarcinogenic and genotoxic potential (see Table S2).

#### 3.1.3. DrugMatrix

The DrugMatrix is a toxicogenomics database, which was obtained and made publicly available by the National Toxicology Program (NTP) from the Gene Expression Omnibus (GEO) [32] with the accession number, GSE57822. It contains gene expression profiles sampled from male Sprague-Dawley rat tissue (liver, kidney, heart and thigh muscle) and cultured rat hepatocytes after single and repeated dosage treatment with 376 chemicals, with control samples from male rats kept in equal conditions. Chemicals were administered in different doses and for different durations (ranging from 6 h to 7 days), and each combination of tissue, chemical, dosage and duration was replicated with three animals, leading to 5587 gene expression profiles. In male rat liver, only 200 of 376 chemicals were profiled, resulting in 654 different combinations of chemical, dosage and duration and 1939 expression profiles. The gene expression profiles were profiled using the Affymetrix Rat Genome 230 2.0 Array. Through CPDB, hepatocarcinogenicity and genotoxicity annotations are available for 132 of the 200 compounds profiled in male rat liver, which translates to 440 conditions with known hepatocarcinogenic and genotoxic potential (see Table S3).

#### 3.1.4. Comparison of TG-GATEs and DrugMatrix

Fifty-one chemicals were profiled by both TG-GATEs and DrugMatrix. For the overlapping chemicals, the dose levels used in DrugMatrix were generally higher than the ones used in TG-GATEs. The dose levels selected for the TG-GATEs repeat dosage experiments were considered to be acceptable for 1-month repeated dosing [8]. The DrugMatrix doses were selected based on estimates of the maximum tolerated dose and fully effective dose generated from literature research and preliminary dose finding studies [33].

### 3.2. Data Preprocessing

TG-GATEs data were normalized with robust multi-array average (RMA) normalization using the R Bioconductor package affy [34]. RMA normalized data from DrugMatrix were downloaded from the DrugMatrix FTP server [35]. For all conditions in the two datasets, log2 intensity ratios were calculated for each probe set as the difference in the average log2 intensity observed in treated samples and controls. Affymetrix probe set identifiers were mapped to official gene symbols using the Bioconductor package biomaRt for R [36]. The expression values of probe sets mapping to the same gene symbol were averaged. Differentially expressed genes were identified for two commonly used intensity ratio cutoffs, 1.5-fold and 2-fold up- or down-regulation. These gene fingerprints were stored for each condition.

### 3.3. Pathway Enrichment

Gene symbols were mapped to corresponding *Rattus norvegicus* pathways obtained from the KEGG database [10]. For each pathway in the KEGG database, a hypergeometric test was performed to check for significant pathway perturbation. The *p*-value is computed as:

(1) $$P(X\geq m)=\sum_{i=m}^{M}\frac{\left(\begin{array}{c} M\\ m \end{array}\right)\left(\begin{array}{c} N-M\\ n-m \end{array}\right)}{\left(\begin{array}{c} N\\ n \end{array}\right)}$$

where *N* is the number of all genes for which gene expression was measured, *M* is the number of genes in the pathway of interest, *n* is the number of differentially expressed genes and *m* is the number of differentially expressed genes that are part of the pathway of interest. The resulting *p*-values were corrected for multiple hypothesis testing with Benjamini–Hochberg correction [37].

### 3.4. Similarity Scoring

The most commonly used similarity measures for gene expression profiles are the Pearson correlation and the Euclidean distance [32]. However, the number of differentially expressed genes is small compared to the total number of genes that were profiled. This leads to sparse gene fingerprints. Therefore, both methods were deemed not applicable for measuring the similarity of gene fingerprints.

In chemoinformatics, fingerprints are used to score the similarity of chemical substances, e.g., the extended connectivity fingerprints (ECFP) [38]. Similarity based on ECFP is computed using the Tanimoto similarity coefficient, which was derived from the Jaccard index (also called the Jaccard similarity coefficient) [39].

The Jaccard index is a similarity measure that is used to define the Jaccard distance, a metric for computing the distance of arbitrary sets. The Jaccard index *J* is defined as the ratio of the number of elements in the overlap of two sets *A* and *B* and the number of elements in the union of the two sets:

(2) $$J=\frac{\left|A\cap B\right|}{\left|A\cup B\right|}$$

This is equivalent to computing:

(3) $$J=\frac{\left|A\cap B\right|}{\left|A|+|B\left|-\right|A\cap B\right|}$$

which does not require the union of the sets *A* and *B*. The Tanimoto coefficient *T* is the equivalent to the Jaccard index defined on binary vectors *X*, *Y* ∈ {0, 1}*^n^*:

(4) $$T=\frac{\sum_{i=1}^{n}X_{i}\wedge Y_{i}}{\sum_{i=1}^{n}X_{i}\vee Y_{i}}$$

The gene fingerprints used for the similarity scoring are not binary vectors, as they present information on upregulated genes (represented as 1), downregulated genes (represented as −1) and non-regulated genes (represented as 0). We defined a modified Tanimoto coefficient *S*, which accounts for the ternary representation. For two gene fingerprints *X*, *Y* ∈ {−1, 0, 1}*^n^*, where *n* is the number of measured genes, the similarity score *S* is defined as:

(5) $$S=\frac{\sum_{g=1}^{n}\delta (X_{g},Y_{g})}{\sum_{g=1}^{n}\left|X_{g}|+|Y_{g}|-\delta (X_{g},Y_{g}\right)}$$

where *δ*(*x,y*) is the Kronecker delta:

(6) $$\delta (x,y)=\{\begin{array}{cc} 1, & x=y\\ 0, & \text{else} \end{array}$$

The modified similarity score *S* allows the scoring of gene fingerprints similarly to the scoring of ECFP fingerprints with the Tanimoto coefficient *T* . If *X* and *Y* are binary vectors, the similarity score *S* and Tanimoto coefficient *T* will be equal.

During the analysis of the scoring schemes, we found that many genes provide little information. This is due to common up- or down-regulation in response to drug administration, regardless of the toxicological outcome. To account for these genes, we further modified the similarity score by introducing a weight vector *w*. Each gene is assigned a weight depending on its frequency in the database:

(7) $$w_{g}=-{\text{log}}_{10}\frac{\sum_{c\in C}\left|c_{g}\right|}{N}$$

where *N* is the number of compounds in the database and *C* is the set of gene fingerprints of the database compounds, *i.e.*, *c_g_*is 1 if gene *g* is upregulated in compound *c*, −1 if *g* is downregulated, and 0 if *g* is not deregulated. The weight *w_g_*corresponds to the negative decadic logarithm of the probability of observing deregulation of gene *g*, when randomly choosing a compound from the database. This concept is commonly used in information theory, where it is known as the information content or self-information [40]. The final similarity coefficient for scoring the similarity of two gene fingerprints is then defined as:

(8) $$S=\frac{\sum_{g=1}^{n}w_{g}\delta (X_{g},Y_{g})}{\sum_{g=1}^{n}w_{g}(|X_{g}|+|Y_{g}|-\delta (X_{g},Y_{g}))}$$

### 3.5. Performance Evaluation

To assess the performance of the similarity scoring, we extracted gene fingerprints from a dataset of gene expression profiles not included in either DrugMatrix or TG-GATEs. The evaluation dataset is publicly available from GEO under the accession number GSE53082 [12]. It contains gene expression profiles for two genotoxic carcinogens, 11 non-genotoxic carcinogens and two non-hepatocarcinogens (see Table 1). Ten of the 15 chemicals are included in one or both of TG-GATEs and DrugMatrix. RMA normalized data were obtained from GEO, and gene fingerprint extraction was performed for each chemical as previously described for TG-GATEs and DrugMatrix. We used our similarity score to extract the most similar conditions in TG-GATEs and DrugMatrix and compared them based on hepatocarcinogenic and genotoxic potential. Enriched KEGG pathways were computed for each chemical.

## Conclusions

We present a new tool for the hepatocarcinogenicity evaluation of drug candidates in rodents. We developed a new similarity scoring method for gene expression profiles that allows robust identification of chemicals with similar hepatocarcinogenic and genotoxic potential. We provide a web application, ToxDBScan, which allows us to perform a similarity search against the two largest publicly available databases in toxicogenomics, TG-GATEs and DrugMatrix, using a newly developed similarity score. ToxDBScan is easy to use and allows a very fast identification of chemicals similar to the query. Since only the deregulated genes are required as input, the tool is independent of the specific microarray or sequencing platform used for transcriptomic profiling. We evaluated the newly developed similarity score with 15 compounds from an experiment not contained in either TG-GATEs or DrugMatrix. We found our scoring system to be capable of robustly identifying compounds with similar hepatocarcinogenic and genotoxic potential. To assess the viability of the similarity score, we performed a classification of the chemicals in the evaluation dataset. All 15 chemicals were assigned to their correct carcinogenicity class. ToxDBScan is publicly available from the ZBIT Bioinformatics Toolbox [11].

## Supplementary Materials

Supplementary materials can be found at http://www.mdpi.com/1422-0067/15/10/19037/s1.

## Supplementary Files

Supplementary File 1
