# Supplementary material for: ToxDBScan: Large-Scale Similarity Screening of Toxicological Databases for Drug Candidates
Source: Int J Mol Sci. 2014 Oct 21;15(10):19037–55. doi: 10.3390/ijms151019037 (PMC4227259; doi:10.3390/ijms151019037)
Supplement: Supplementary File 1 [file ijms-15-19037-s001.zip › ijms-64426-Figure S1.pdf]

Supplementary Information

Figure S1. Gene expression heat maps of similar compounds. For each test chemical, we extracted the most similar chemicals included in either TG-GATEs or DrugMatrix. Each column corresponds to a chemical that was identified as similar. The chemicals are sorted from left to right by descending similarity score. The heat maps show 20 selected genes from the gene fingerprints of the test chemical. The 10 genes above the black line are up-regulated in the test compound, and the 10 genes below are down-regulated. Genes were selected based on average expression in the identified chemicals. The color bar above the chemical name indicates the hepatocarcinogenicity annotation, where blue means non-genotoxic hepatocarcinogen, red means genotoxic hepatocarcinogen and green means non-hepatocarcinogen. Heat maps are shown for (**a**) Acetamide; (**b**) Cerufoxime; (**c**) Direct Black 38; (**d**) Cyproteone acetate; (**e**) Diethylsilbestrol; (**f**) Dehydroepiandrosterone; (**g**) Nitrosodimethylamine; (**h**) Ethionine; (**i**) Methylcarbamate; (**j**) Methapyrilene; (**k**) Nifedipine; (**l**) Phenobarbital; (**m**) Piperonylbutoxide; (**n**) Thioacetamide; and (**o**) Wy-14643.

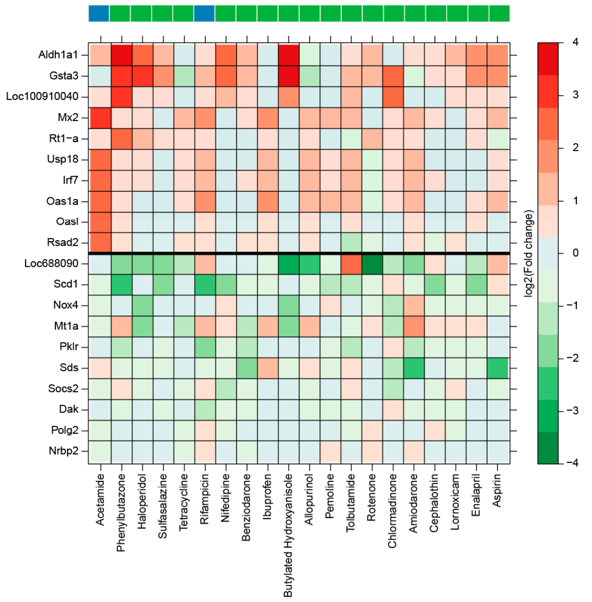

(a) Acetamide (NGC)

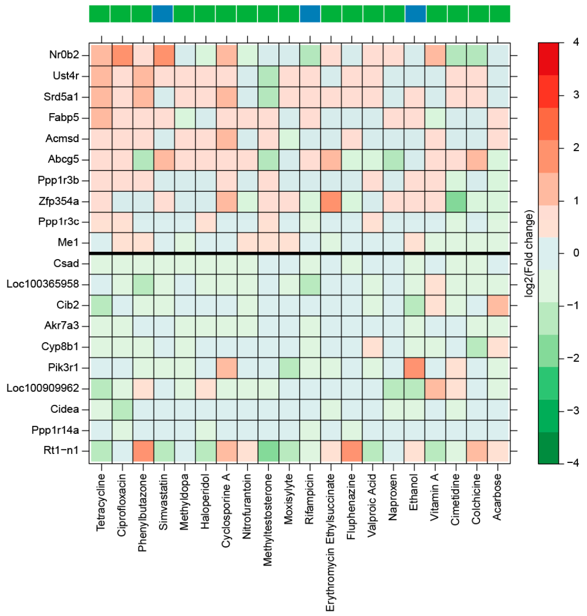

(b) Cerufoxime (NC)

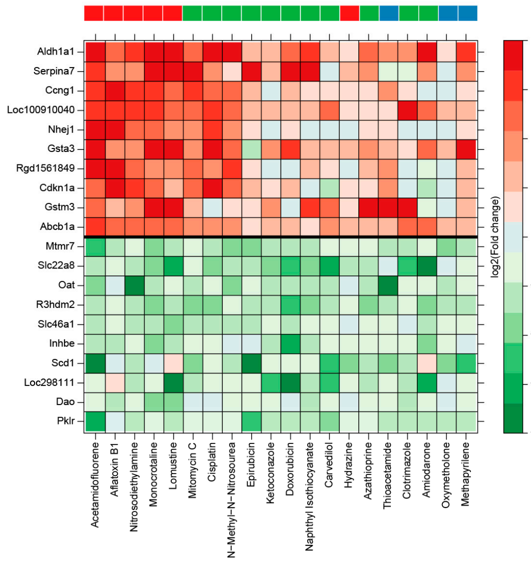

(c) Direct Black 38 (GC)

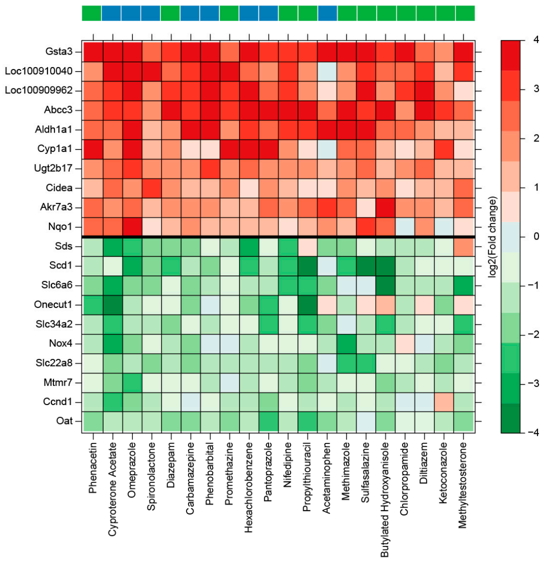

(d) Cyproterone acetate (NGC)

Figure S1. *Cont.*

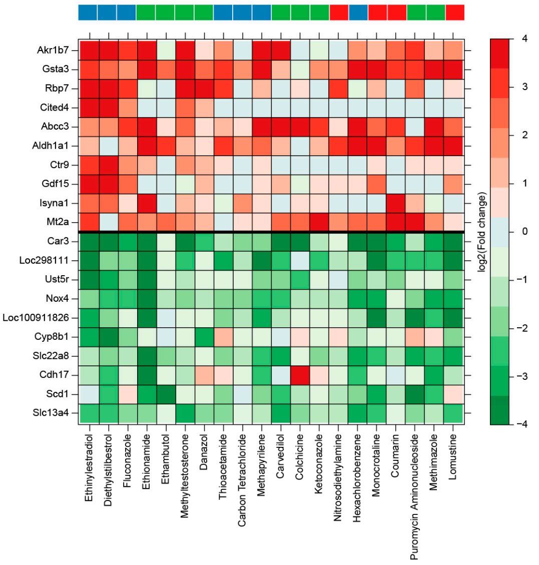

(e) Diethylsilbestrol (NGC)

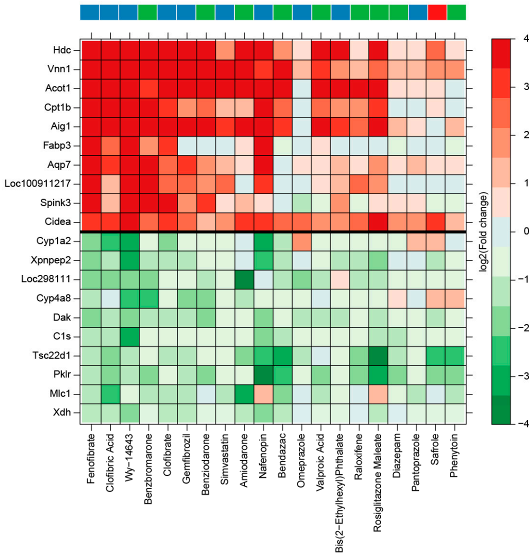

(f) Dehydroepiandrosterone (NGC)

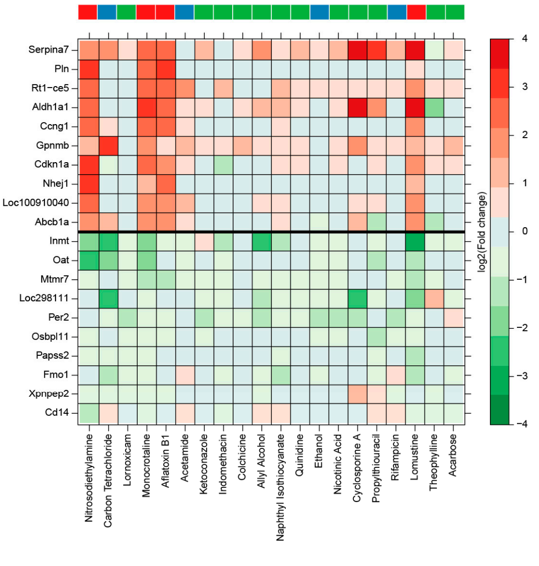

(g) Nitrosodimethylamine (GC)

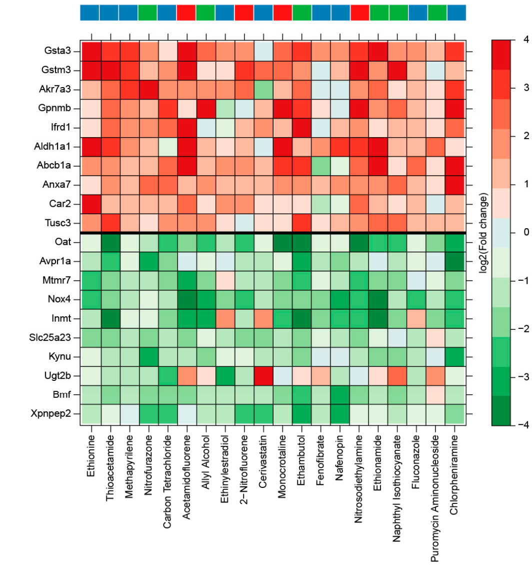

(h) Ethionine (NGC)

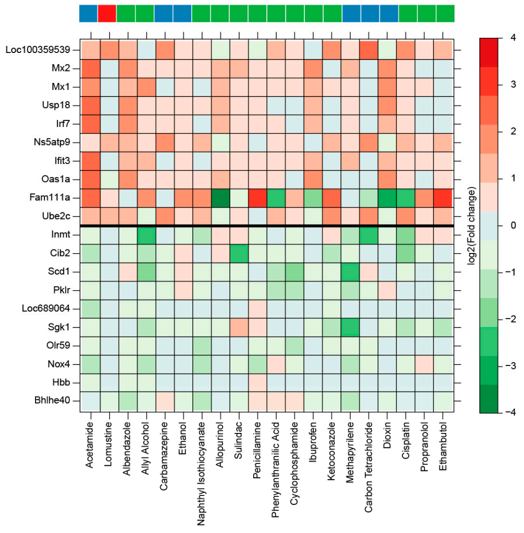

(i) Methylcarbamate (NGC)

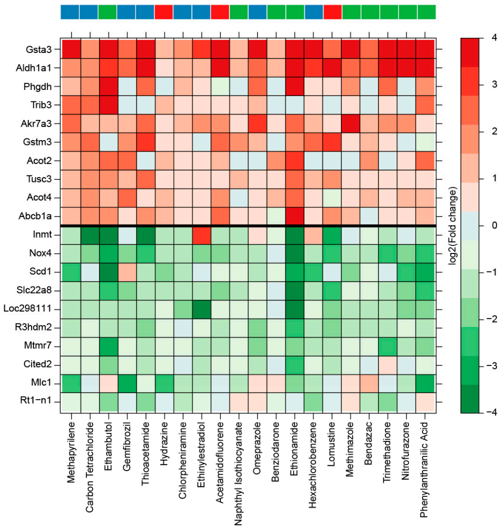

(j) Methapyrilene (NGC)

Figure S1. *Cont.*

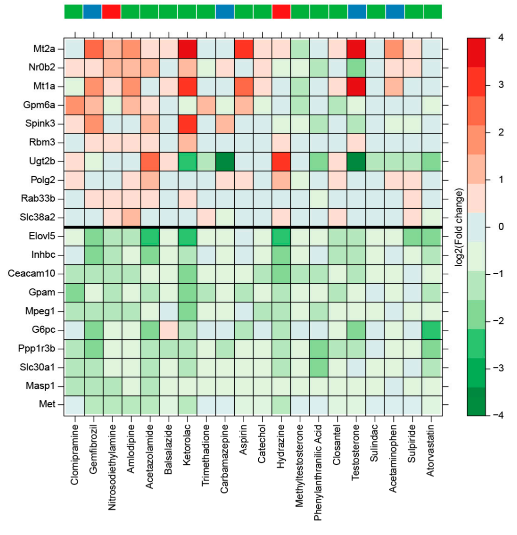

(k) Nifedipine (NC)

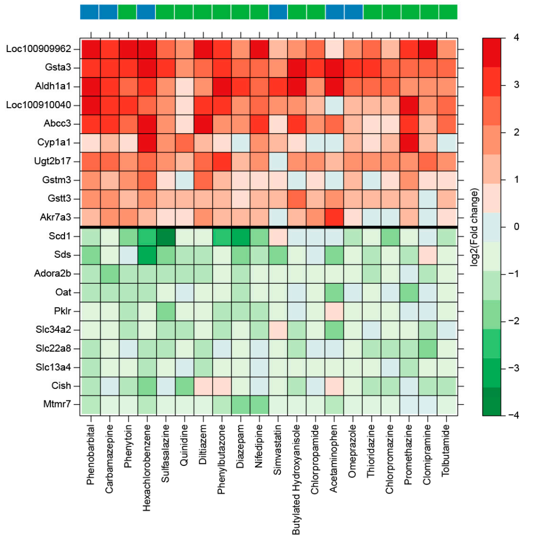

(l) Phenobarbital (NGC)

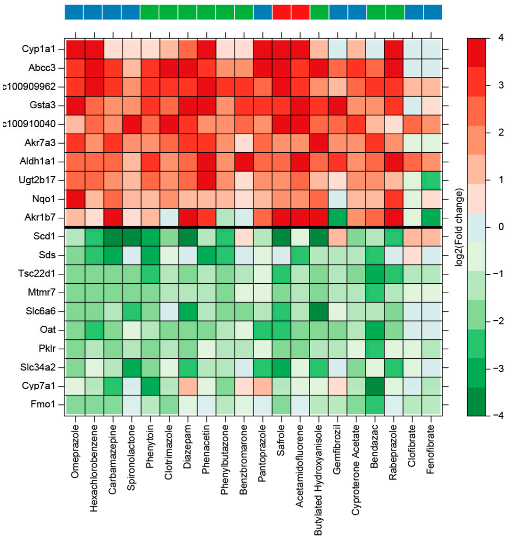

(m) Piperonylbutoxide (NGC)

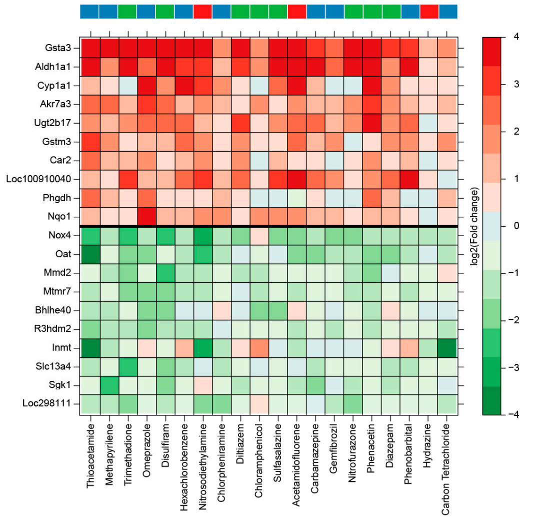

(n) Thioacetamide (NGC)

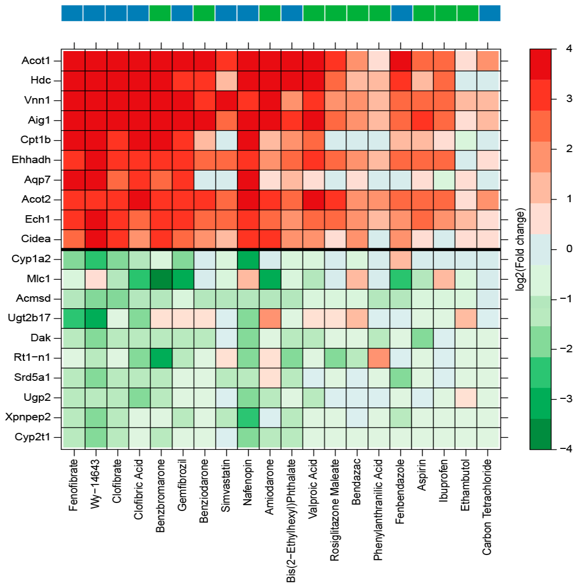

(o) Wy-14643 (NGC)
